# Relationship between Proton Magnetic Resonance Spectroscopy of Frontoinsular Gray Matter and Neurodevelopmental Outcomes in Very Low Birth Weight Children at the Age of 4

**DOI:** 10.1371/journal.pone.0156064

**Published:** 2016-05-25

**Authors:** Wojciech Durlak, Izabela Herman-Sucharska, Andrzej Urbanik, Małgorzata Klimek, Paulina Karcz, Grażyna Dutkowska, Magdalena Nitecka, Przemko Kwinta

**Affiliations:** 1 Department of Pediatrics, Jagiellonian University, Wielicka 265, 30–663 Cracow, Poland; 2 Department of Electroradiology, Jagiellonian University, Michalowskiego 12, 31–126 Cracow, Poland; 3 Department of Radiology, Jagiellonian University, Kopernika 19, 31–501 Krakow, Poland; 4 Department of Applied Psychology and Human Development, Jagiellonian University, Wielicka 265, 30–663 Cracow, Poland; University of New Mexico, UNITED STATES

## Abstract

Very low birth weight is associated with long term neurodevelopmental complications. Macroscopic brain abnormalities in prematurity survivors have been investigated in several studies. However, there is limited data regarding local cerebral metabolic status and neurodevelopmental outcomes. The purpose of this study was to characterize the relationship between proton magnetic resonance spectra in basal ganglia, frontal white matter and frontoinsular gray matter, neurodevelopmental outcomes assessed with the Leiter scale and the Developmental Test of Visual Perception and selected socioeconomic variables in a cohort of very low birth weight children at the age of four. Children were divided in three groups based on the severity of neurodevelopmental impairment. There were no differences in spectroscopy in basal ganglia and frontal white matter between the groups. Lower concentrations of N-acetylaspartate (NAA), choline (Cho) and myoinositol (mI) were observed in the frontoinsular cortex of the left hemisphere in children with neurodevelopmental impairment compared to children with normal neurodevelopmental outcomes. Higher parental education, daycare attendance and breastfeeding after birth were associated with more favorable neurodevelopmental prognosis, whereas rural residence was more prevalent in children with moderate and severe impairment. Our study demonstrates the role of long term neurometabolic disruption in the left frontoinsular cortex and selected socioeconomic variables in determination of neurodevelopmental prognosis in prematurity survivors.

## Introduction

The annual rate of preterm birth in the United States of America is 11,39% [1] and in Poland 6,3%. Even though a gradual decline has been observed in the recent years, the long-term morbidity is still high. Particularly, very low birth weight is associated with neurodevelopmental complications. Advances in obstetric and neonatal care have increased survivor rates among extremely premature children, but the percentage of long term complications including cognitive impairment, bronchopulmonary dysplasia (BPD), retinopathy of prematurity (ROP) remains significant, particularly in extremely prematurely born children. The mechanisms underlying brain injury in very premature children have been described.[2] It has been suggested that even children born prematurely who maintain normal intellectual performance are at increased risk of long term executive dysfunction, including deficits of attention or visuospatial processing.[3] Therefore a better characterization of cerebral neurometabolic status in preschool children with a history of prematurity might facilitate early diagnosis of neurodevelopmental dysfunction and help develop a clinically useful marker.

Chronic neurologic sequelae of prematurity in children without significant cerebral structural abnormalities have been poorly understood. Proton magnetic resonance spectroscopy (1H-MRS) is a non-invasive technique which provides insight into the brain metabolism. It allows to measure specific metabolite concentrations in the cerebral tissue, including N-acetylaspartate (NAA), choline (Cho), creatine (Cr), myoinositol (mI). There are only a few reports from studies using 1H-MRS in preterm children, most of them assessing cerebral metabolic status at term or near-term and trying to develop a prognostic tool for neurodevelopmental outcomes. Hart et al.[4] found altered NAA levels in the posterior white matter at term to be a potential marker for later neurodevelopmental outcome. Similarly, Kendall et al. discovered that the combination of Cho/Cr and NAA/Cho concentrations in the posterior white matter measured at term might help predict motor development at 1 year of age. Interestingly the group of van Kooij et al.[5] has shown that there might a relationship between cerebellar neurometabolite concentration at term and neurodevelopmental outcomes at the age of 2. There are two studies by Bathen [6] and Gimenez [7] evaluating adolescents with the history of prematurity using cerebral 1H-MRS. To our knowledge there is also only one study performed in a group of preschool children that found significant neurometabolic differences between VLBW children compared to term controls, but only in the white matter. [8] The authors found a significant difference in metabolite concentrations in the white matter of the frontal lobe.

Therefore, we performed a cross sectional study of 1H-MRS and neurodevelopmental assessment in a cohort of VLBW children at the age of 4. Our primary aim was to characterize the neurometabolic status at specific locations both in gray and white matter and its relationship with the results of two cognitive tests. Furthermore, we wanted to investigate whether 1H-MRS might be useful to help predict neurodevelopmental outcomes in the preschool VLBW children.

## Methods

The current project was planned as a follow-up of the study conducted between June 2008 and April 2011, whose main aim was to evaluate the role of genetic and biochemical factors in the development of late complications of prematurity. Each child was a subject to meticulous and systematic observation. The presence of BPD, ROP and periventricular leukomalacia (PVL) were recorded. Several tests were performed, including: a full head ultrasound with the Doppler’s evaluation (every 7 days), a detailed ophthalmological assessment (every 2 weeks), and a physiologic test evaluating oxygen requirement (the 36th week of postmenstrual age). Thus, the perinatal health status of the described population was well defined and well documented. All children who are alive at the age of 4 years were invited to participate in the follow-up study (n = 101). The following inclusion criteria have been used: birthweight < 1500g, gestational age <32 weeks, age at follow-up 3,5–4,5 years. The children with multiple congenital defects have been excluded from the study. The study had been approved by the Ethics Committee of Jagiellonian University, Faculty of Medicine.

It was conducted in the Follow-up Pediatric Department of the Polish-American Children’s Hospital. The patients were recruited between September 2012, and April 2015. After signing the informed consent by the parents, detailed psychomotor development evaluation, Magnetic Resonance Imaging (MRI), and 1H-MRS were performed in all children. Parents were asked to fill in questionnaires regarding the socioeconomic status of the family and a questionnaire assessing the past and the present health status of the child.

### Psychomotor development

The neurodevelopmental assessment was conducted with the following examination tools:

- Leiter scale–it is a non-verbal psychometric evaluation containing 52 tests. The scale is designed for children from 3 to 15 years of age. It is an individual test and tasks are aimed at engaging the child. It is the only standardized test for children aged 4 years in Poland. The results are presented as an Intelligence quotient (IQ) score. The mean value of the test in the population is equaled to 100 points, and standard deviation (SD) is equaled to 15 points. The abnormal result was defined as a result below 85 points.
- Developmental test of Visual Perception (DTVP)—Visual perceptive skills were tested using the DTVP-3, the most recent revision of the classic Marianne Frostig DTVP. Five subtests were used in all children. In the Eye-Motor Coordination Test, patients were asked to draw straight or curved lines according to given boundaries. The Figure-ground Test aimed at isolation of simple, defined figures hidden in an increasingly complex background. In the Constancy of Shape Test, children were asked to find as many partially covered figures as possible. During the Position in Space Test, a stimulus figure was shown to the child who had to choose a corresponding or different one from a series of figures. Finally, in the Spatial Relationships Test, children were shown an increasingly complex line of arrangements and asked to copy them. The result of the test consists of overall score and five partial results.[9] DTVP has been validated and is considered internally consistent when compared to other established tools assessing visual perception, such as Beery-Buktenica Developmental Test of Visual-Motor Integration (VMI) and Test of Visual Perceptual Skills (TVPS-3).[10] The results yield scores including raw values, age equivalents, percentiles, composite quotients that provide insight into the general visual perceptual abilities, as well as, indicate specific strengths and weaknesses. The abnormal result was set below 85 points.

### Magnetic resonance imaging

Children were subjected to MRI studies using a 1,5T GE HDxt system (General Electric Healthcare, Milwaukee, WI, USA) soft version 16.00. All MRI and MRS were equipped with an 8-channel head coil. Morphological brain changes were assessed using standard sequences:

1. Propeller T2 fast spin echo sequence in axial plane (slice thickness 4,0mm, spacing 2,0mm, TR 6000ms, TE 97ms, FOV 24cm, matrix 320x320)
2. T2 FRFSE-XL fast spin echo sequences in sagittal plane (slice thickness 4,0mm, spacing 2,0mm TR 3660ms, TE 88ms, FOV 24cm, matrix 384x224)
3. T2 FRFSE-XL fast spin echo sequences in coronal plane (slice thickness 4,0mm, spacing 2,0mm TR 4600ms, TE 88ms, FOV 24cm, matrix 384x224)
4. Propeller T2 FLAIR in axial plane (slice thickness 4,0mm, spacing 2,0mm, TR 8000ms, TE 123ms, T1 8000ms, FOV 24cm, matrix 288x288)
5. T1 spin echo sequence in axial plane (slice thickness 4,0mm, spacing 2,0mm, TR 320ms, TE 9ms, FOV 24cm, matrix 512x224)
6. GRE T2* gradient echo sequence in axial plane (slice thickness 4,0mm, spacing 2,0mm, TR 720ms, TE 15ms, flip angle 20, FOV 24cm, matrix 320x192)
7. FSPGR T1 gradient echo IR prepared sequence in axial, coronal and sagittal plane (slice thickness 2,0mm, spacing -1,0mm, TR 10ms, TE 4,4ms, TI 450ms, flip angle 12, FOV 20cm, matrix 320x192)
8. DWI echo planar imaging (DWEPI) sequence in axial plane (slice thickness 4,0mm, spacing 2,0mm, TR 8000ms, TE 98ms, FOV 24cm, matrix 128x128)

### Magnetic resonance spectroscopy

**Fig 1:**
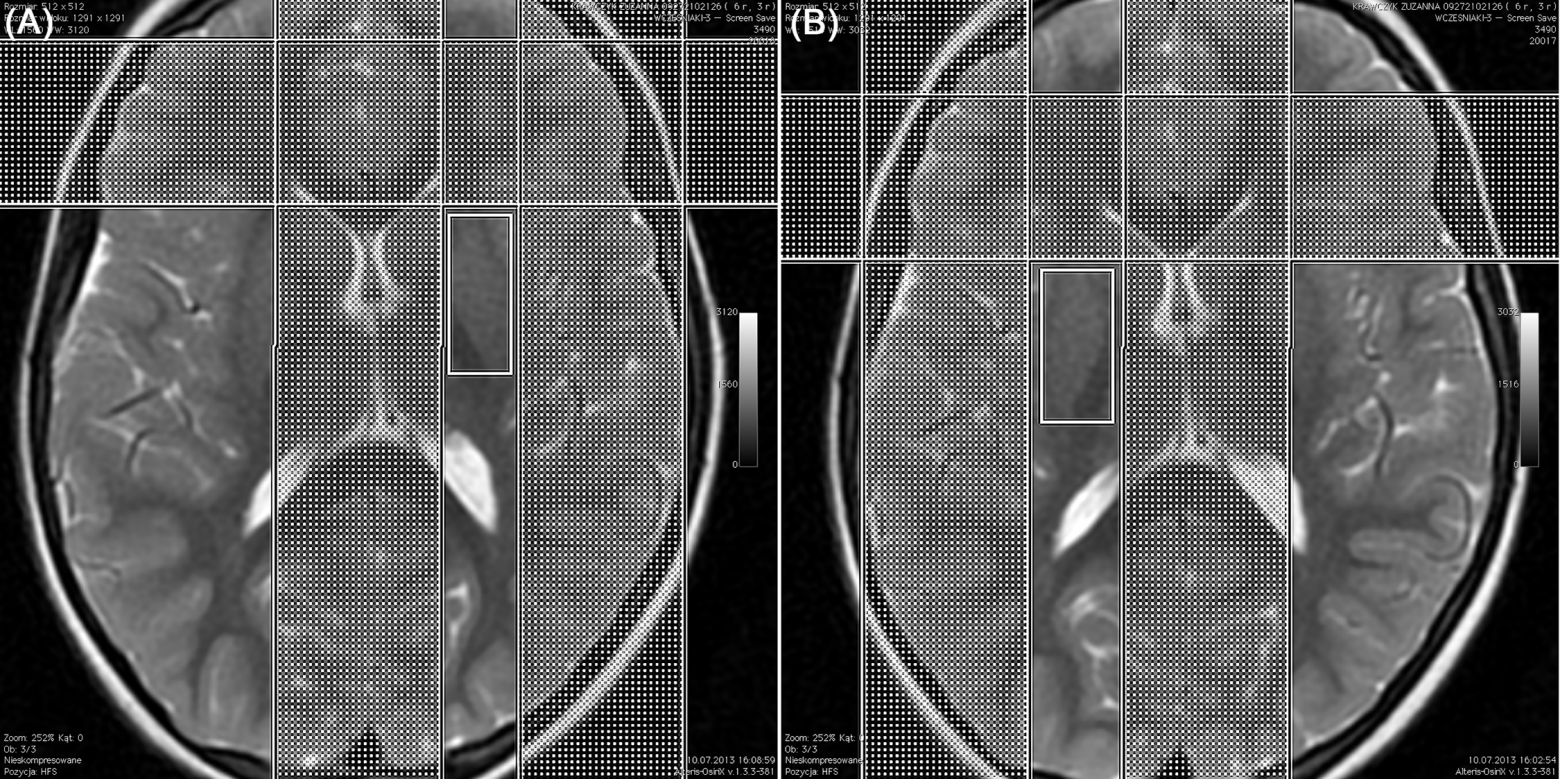
Brain 1H-MRS, PRESS technique, frFSET2 sequence, axial plane: voxel localized in the left (A) and right (B) basal ganglia.

**Fig 2:**
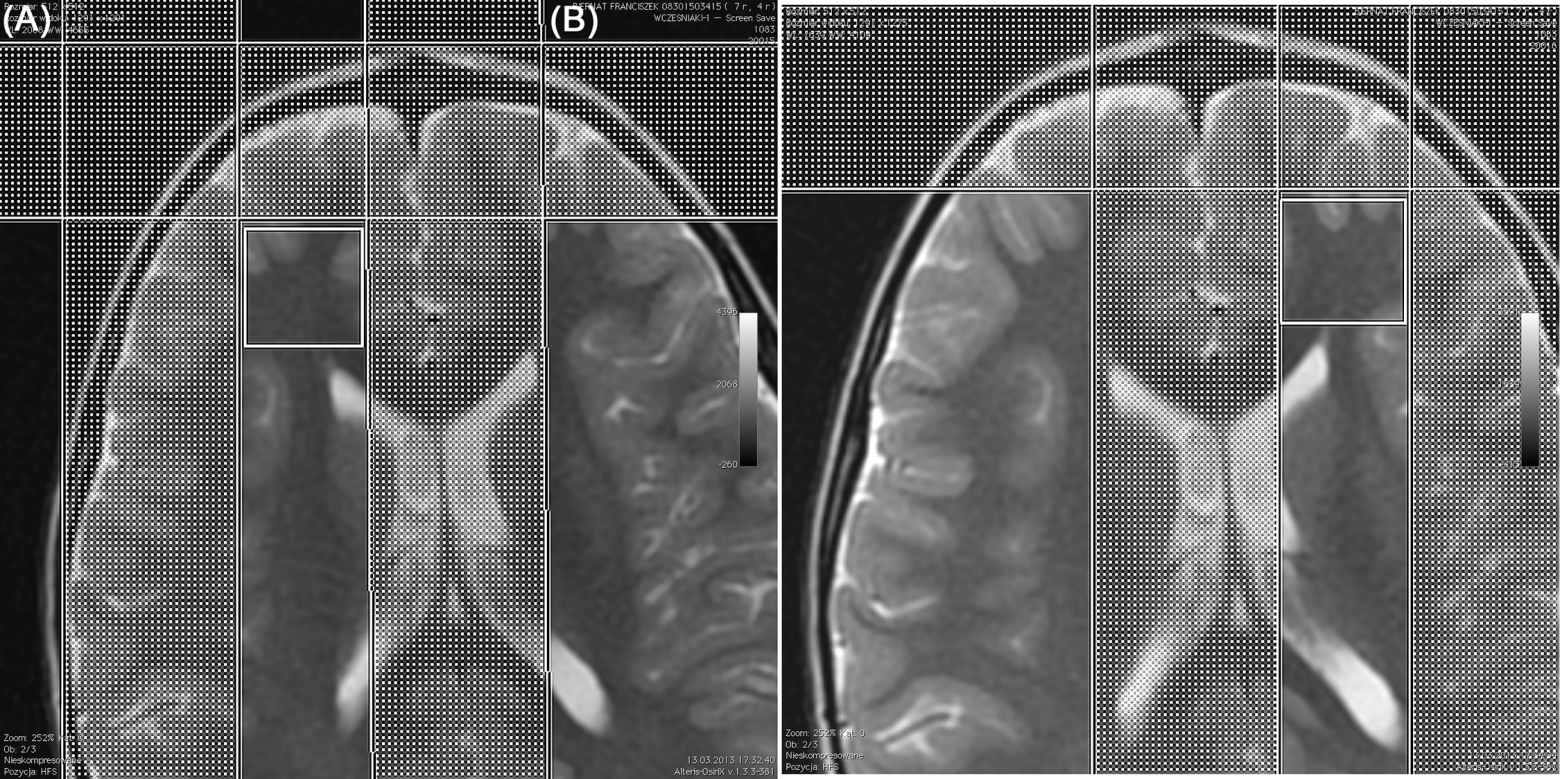
Brain 1H-MRS, PRESS technique, frFSET2 sequence, axial plane: voxel localized in the right (A) and left (B) frontal lobe white matter.

**Fig 3:**
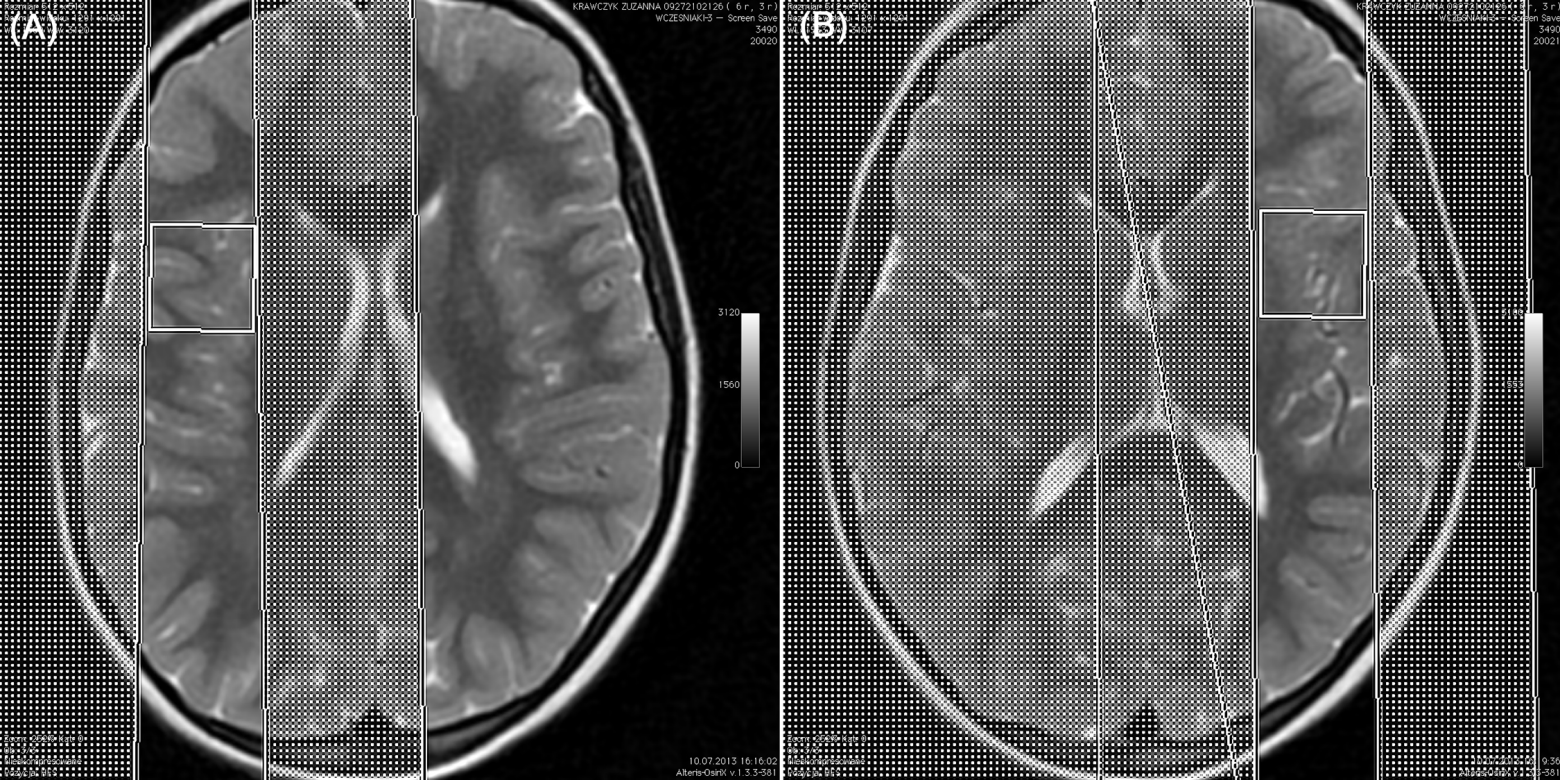
Brain 1H-MRS, PRESS technique, frFSET2 sequence, axial plane: voxel localized in the right (A) and left (B) frontoinsular gray matter.

1H-MRS was performed using single-voxel spectroscopy technique (SVS). The spectrum was acquired in less than 5 minutes, using the automated PROBE (PROton Brain Examination) spectroscopy package. Spectrum was generated after 64 acquisitions with the localization sequences point-resolved spectroscopy (PRESS) with TR = 1500ms, TE = 35ms. The PRESS sequence utilizes a 900 and two 1800 radiofrequency pulse. For water suppression CHESS sequence (CHEmical shift Selective imaging Sequence) was used with a frequency-selective 900 pulse to selectively excite the water signal, followed by a dephasing gradient. After acquisition, the 1H-MRS data were analyzed on an AW (Adventage 145 Workstation, GE) 4.5 workstation with SAGE (Spectroscopy Analysis by GE). The following metabolites were manually selected from the spectrum: lipids (0,9–1,0 ppm), lactates (1,33 ppm), NAA (2,02 ppm), Cr (3,02 ppm), Cho (3,22 ppm), and mI (3,56 ppm). Metabolism of the central nervous system was investigated at the following locations: 1 –frontoinsular gray matter, 2 –basal nuclei, 3 –white matter of the frontal lobes. Fig 1, Fig 2 and Fig 3 present details regarding voxel locations. The volume of interest (VOI) was approximately 6 cm^3^. The size of VOI was adjusted to the anatomical size of the area the spectrum was collected from.

The acquired spectra were analyzed for changes in the relative concentrations of the following metabolites: lipids/creatine (Lip/Cr), lactates/creatine (Lac/Cr), NAA/Cr, Cho/Cr, and mI/Cr.

### Selection of the groups

Based on the results of the psychomotor evaluation, the children were divided into the following groups:

- normal development–normal results of both tests
- mild neurodevelopmental problems–normal result of the Leiter scale, abnormal result of DTVP
- moderate/severe neurodevelopmental problems–abnormal results of both Leiter scale and DTVP

### Outcome variables

Primary outcome variables were results of the single voxel spectroscopy: ratios of the NAA/Cr, Cho/Cr, mI/Cr, NAA/Cho. Moreover, the correlations between the results of neurodevelopmental tests and metabolites concentrations were evaluated.

### Statistical analysis

The ANOVA was used to determine whether there were any significant differences between the mean values in the three groups. When an overall significant difference in the groups mean values was detected the post-hoc tests were utilized. If the data met the assumption of homogeneity of variances, the Tukey's honestly significant difference test was used, otherwise the Games- Howell test was used. Post-hoc tests were run to confirm the differences between the specific groups. Chi-squared test was used to compare categorical variables.

The study compared multiple 1H-MRS variables and correction for multiple tests was performed. The Sidak’s adjustment for correlated variables was used. Corrected alpha level based on mean correlation factor 0.281 between different measurements equaled 0.015. Based on this calculation, if the unadjusted p value was <0.015, the risk of type I error was <5%. Finally, statistical significance in the presented paper for 1H-MRS measurements was defined at the 2-sided p = 0.015 level and for other analyses at the 2-sided p = 0.05 level. Data was analyzed using SPSS Software (version 22, 2013 by IBM Corporation, Armonk, NY, USA).

## Results

Sixty-five children with a mean birth weight of 1027 g (SD 267g) were evaluated at the mean age of 49 months (range 45–54). Based on the results of neurodevelopmental assessment, 41 children were classified as normal, 14 children were included into the group of mild neurodevelopmental problems and 10 children into the group of moderate/severe neurodevelopmental problems. The comparison of selected demographic and clinical variables between the groups is shown in Table 1. There were no significant differences in age, gender, birth weight and gestational age between the groups. Neither, have we found significant differences in the prevalence of major clinical sequelae of prematurity. Periventricular leukomalacia (PVL) was the only factor with statistically higher prevalence among children with moderate to severe neurodevelopmental problems, but its overall prevalence in the whole cohort was low.

**Table 1:** Comparison of selected demographic and clinical variables between the groups.

Psychomotor development	Normal (n = 41)^a^	Mild (n = 14)^a^	Moderate/severe(n = 10)^a^	P value
Demographic variables				
Female	21 (51%)	6 (43%)	2 (20%)	0.2^b^
Birth weight (g); median (range)	1070 (600–1480)	865 (595–1440)	850 (570–1390)	0.12^c^
Gestational age (weeks); median (range)	28 (24–32)	28 (23–31)	27 (22–31)	0.66^c^
Vaginal delivery	12 (29%)	6 (43%)	5 (50%)	0.38^b^
Multiple pregnancy	8 (20%)	3 (21%)	1 (10%)	0.75^b^
Surfactant administration	25 (61%)	7 (50%)	9 (90%)	0.12^b^
Patent Ductus Arteriosus treatment	5 (12%)	5 (36%)	3 (30%)	0.14^b^
Oxygen at 28^th^ day of life	25 (61%)	12 (86%)	16 (60%)	0.22^b^
Oxygen at 36 weeks postmenstrual age	7 (17%)	6 (43%)	2 (20%)	0.14^b^
Intraventricular haemorrhage grade III or IV	3 (7%)	2 (14%)	3 (30%)	0.31^b^
Periventricular leukomlacia	1 (2%)	3 (21%)	3 (30%)	0.02^b^^,^ ^d^^,^
Retinopathy of prematurity	12 (29%)	8 (57%)	4 (40%)	0.17^b^

^a^ data are presented as number (percentage) of patients unless otherwise indicated.

^b^ p value for chi-squared test.

^c^ p for Kruskall-Wallis ANOVA.

^d^ because ANOVA was significant post-hoc analysis was performed which revealed significant differences between the groups: normal development vs. moderate/severe neurodevelopmental problems.

Selected socioeconomic variables and anthropometric measurements are compared in Table 2. Children with neurodevelopmental impairment, both mild and moderate to severe, were significantly smaller with regards to height, weight and head circumference. We found significant differences in socioeconomic variables. Children without neurodevelopmental problems were more likely to attend daycare and their fathers were more likely to have obtained academic education. More children in this group received breastfeeding after birth. Significantly more children with abnormal neurodevelopmental assessment lived in rural areas.

**Table 2:** Comparison of selected variables recorded at the age of 4 years between the groups.

	Normal (n = 41)^a^	Mild (n = 14)^a^	Moderate/severe (n = 10)^a^	P value
Socioeconomic variables				
Rural residence	26 (63%)	3 (21%)	8 (80%)	0.01^b^^,^ ^d^^,^ ^f^
Maternal education (low/middle/high)	14/18/9	6/4/4	6/4/0	0.2 ^b^
Father’s education (low/middle/high)	18/18/5	9/1/4	4/6/0	0.044 ^b^
Mother without occupation	30 (73%)	11 (79%)	10 (100%)	0.18 ^b^
Father without occupation	2 (5%)	0	0	0.55 ^b^
Sibling at home	28 (68%)	10 (71%)	8 (80%)	0.5 ^b^
Breast milk feeding	20 (49%)	5 (36%)	0	0.01 ^b^^,^ ^e^
Daycare attendance	26 (63%)	5 (36%)	1 (10%)	0.01 ^b^^,^ ^e^
Anthropometric measurements				
Age at evaluation (years); mean (SD)	4.08 (0.34)	4.10 (0.37)	4.12 (0.36)	0.9 ^c^
Height (cm); mean (SD)	101 (3.9)	97 (4.4)	99 (6.0)	0.01 ^c^^,^ ^d^
Height (z-score); mean (SD)	0.04 (1.0)	-1.1 (1.1)	-0.7 (1.3)	0.01 ^c^^,^ ^d^^,^ ^e^
Weight (kg); mean (SD)	15.3 (2.6)	13.3 (1.8)	13.5 (2.3)	0.02 ^c^^,^ ^d^^,^ ^e^
Weight (z-score); mean (SD)	-0.56 (1.3)	-1.78 (1.3)	-1.78 (1.4)	0.01 ^c^^,^ ^d^^,^ ^e^
Head circumference (cm); mean (SD)	49.8 (1.7)	48.7 (2.9)	48 (2.1)	0.02 ^c^^,^ ^e^
Head circumference (z-score); mean (SD)	-1.35 (1.1)	-2.3 (2.1)	-2.8 (1.4)	0.01 ^c^^,^ ^d^^,^ ^e^
	Normal (n = 41)^a^	Mild(n = 14)^a^	Moderate/severe(n = 10)^a^	P value
Socioeconomic variables				
Rural residence	26 (63%)	3 (21%)	8 (80%)	0.01^b^^,^ ^d^^,^ ^f^
Maternal education (low/middle/high)	14/18/9	6/4/4	6/4/0	0.2 ^b^
Father’s education (low/middle/high)	18/18/5	9/1/4	4/6/0	0.044 ^b^
Mother without occupation	30 (73%)	11 (79%)	10 (100%)	0.18 ^b^
Father without occupation	2 (5%)	0	0	0.55 ^b^
Sibling at home	28 (68%)	10 (71%)	8 (80%)	0.5 ^b^
Breast milk feeding	20 (49%)	5 (36%)	0	0.01 ^b^^,^ ^e^
Daycare attendance	26 (63%)	5 (36%)	1 (10%)	0.01 ^b^^,^ ^e^
Anthropometric measurements				
Age at evaluation (years); mean (SD)	4.08 (0.34)	4.10 (0.37)	4.12 (0.36)	0.9 ^c^
Height (cm); mean (SD)	101 (3.9)	97 (4.4)	99 (6.0)	0.01 ^c^^,^ ^d^
Height (z-score); mean (SD)	0.04 (1.0)	-1.1 (1.1)	-0.7 (1.3)	0.01 ^c^^,^ ^d^^,^ ^e^
Weight (kg); mean (SD)	15.3 (2.6)	13.3 (1.8)	13.5 (2.3)	0.02 ^c^^,^ ^d^^,^ ^e^
Weight (z-score); mean (SD)	-0.56 (1.3)	-1.78 (1.3)	-1.78 (1.4)	0.01 ^c^^,^ ^d^^,^ ^e^
Head circumference (cm); mean (SD)	49.8 (1.7)	48.7 (2.9)	48 (2.1)	0.02 ^c^^,^ ^e^
Head circumference (z-score); mean (SD)	-1.35 (1.1)	-2.3 (2.1)	-2.8 (1.4)	0.01 ^c^^,^ ^d^^,^ ^e^

^a^ data are presented as number (percentage) of patients unless otherwise indicated.

^b^ p value for chi-squared test.

^c^ p value for One-way ANOVA.

^d^ because ANOVA was significant post-hoc analysis was performed which revealed significant differences between the groups: normal development vs. mild neurodevelopmental problems.

^e^ because ANOVA was significant post-hoc analysis was performed which revealed significant differences between the groups: normal development vs. moderate/severe neurodevelopmental problems.

^f^ because ANOVA was significant post-hoc analysis was performed which revealed significant differences between the groups: mild neurodevelopmental problems vs. moderate/severe neurodevelopmental problems.

Comparison of neurodevelopmental evaluation results is presented in Table 3. The results of Leiter scale significantly differed between all three groups. The results of DTVP test were significantly lower in children with mild or moderate/severe neurodevelopmental problems than in the control group.

**Table 3:** Comparison of neurodevelopmental assessment at the age of 4 years between the groups (data are presented as mean and standard deviation).

Psychomotor assessment	Normal (n = 41)	Mild (n = 14)	Moderate/severe (n = 10)	p for ANOVA	Normal vs mild^a^	Normal vs moderate/severe^a^	Mild vs moderate/severe^a^
Leiter test	106 (11)	93 (8.4)	64(10)	0.001	0.01	0.001	0.001
DTVP	100 (10)	74 (13)	66 (8.1)	0.001	0.001	0.001	0.164

^a^ post-hoc analysis.

1H-MRS variables obtained in the studied groups are presented in Table 4. The precise voxel locations are presented in Fig 1, Fig 2 and Fig 3. We found no differences in NAA/Cr, Cho/Cr and mI/Cr concentrations in frontal white matter, basal ganglia and right frontoinsular cortex between the groups. The ratios to creatine concentration for all three metabolites were higher in the left frontoinsular cortex in children with normal results of Leiter scale and DTVP. There was a linear relationship between Cho/Cr concentration and severity of neurodevelopmental impairment measured in Leiter scale (r = 0.412; p = 0.001) and Frostig scale (r = 0.261; p = 0.04). Moreover, the correlation between mI/Cr and Frostig test results was observed (r = 0.256; p = 0.05). There were no differences in Lip/Cr and Lac/Cr concentrations in all locations.

**Table 4:** Primary 1H-MRS outcome variables in the studied groups.

	Normal (n = 41)^a^	Mild (n = 14)^a^	Moderate/severe (n = 10)^a^	Unadjusted P value^b^	Adjusted P value^c^
Left frontoinsular gray matter					
NAA/Cr	1.86 (0.25)	1.73 (0.19)	1.66 (0.21)	0.04 ^e^	0,12
Cho/Cr	0.96 (0.13)	0.93 (0.14)	0.81 (0.13)	0.01 ^e^	0,04
mI/Cr	0.81 (0.11)	0.74 (0.08)	0.73 (0.11)	0.05 ^d^^,^ ^e^	0,14
Right frontoinsular gray matter					
NAA/Cr	1.78 (0.19)	1.72 (0.28)	1.78 (0.23)	0.62	0,88
Cho/Cr	0.9 (0.13)	0.91 (0.17)	0.82 (0.2)	0.34	0,5
mI/Cr	0.76 (0.11)	0.74 (0.09)	0.73 (0.13)	0.55	0,65
Left frontal white matter					
NAA/Cr	1.81 (0.32)	1.69 (0.27)	1.89 (0.39)	0.27	0,35
Cho/Cr	0.91 (0.14)	0.87 (0.19)	0.94 (0.18)	0.47	0,6
mI/Cr	0.74 (0.16)	0.72 (0.14)	0.77 (0.14)	0.7	0,85
Right frontal white matter					
NAA/Cr	1.74 (0.25)	1.89 (0.26)	1.85 (0.17)	0.1	0,22
Cho/Cr	0.92 (0.17)	0.95 (0.17)	0.96 (0.11)	0.61	0,8
mI/Cr	0.76 (0.13)	0.80 (0.13)	0.83 (0.18)	0.32	0,45
Left basal ganglia					
NAA/Cr	1.48 (0.2)	1.39 (0.19)	1.56 (0.13)	0.1	0,22
Cho/Cr	0.77 (0.14)	0.76 (0.1)	0.86 (0.19)	0.13	0,25
mI/Cr	0.58 (0.12)	0.55 (0.1)	0.67 (0.15)	0.06	0,15
Right basal ganglia					
NAA/Cr	1.50 (0.18)	1.51 (0.18)	1.42 (0.17)	0.4	0,55
Cho/Cr	0.74 (0.14)	0.8 (0.11)	0.72 (0.13)	0.24	0,33
mI/Cr	0.55 (0.13)	0.55 (0.11)	0.49 (0.09)	0.32	0,45

^**a**^ data are presented as mean and standard deviation (SD).

^b^p value for One-way ANOVA (unadjusted).

^c^ p-value adjusted for multiple comparisons.

^d^ because ANOVA was significant, post-hoc analysis was performed which revealed significant differences between the groups: normal development vs. mild neurodevelopmental problems.

^e^ because ANOVA was significant, post-hoc analysis was performed which revealed significant differences between the groups: normal development vs. moderate/severe neurodevelopmental problems.

## Discussion

This study investigated the relationship of brain tissue metabolite concentration at specific locations and selected neurodevelopmental outcomes in VLBW children at the age of 4 years. Our cohort of VLBW children was divided into three subgroups based on the results of Leiter scale and DTVP (normal, mild neurodevelopmental abnormalities, moderate to severe neurodevelopmental abnormalities). We discovered a statistically significant difference in NAA/Cr, Cho/Cr and mI/Cr ratios in the frontoinsular gray matter of the left hemisphere. Moreover, we observed a linear relationship between metabolite concentration and severity of neurodevelopmental impairment. The highest concentrations were observed in children without developmental impairment and the lowest in children with moderate andsevere neurodevelopmental delay. There were no similar results in the right hemisphere.

The insula and the anterior insular cortex (AIC) in particular are believed to be a multimodal integration center[11]. This region has been implicated in voluntary action coordination[12] and perception, including visual awareness, decision making[13,14] and time perception.[15] AIC activation has been very commonly reported in functional MRI (fMRI) studies evaluating cognitive skills. To our knowledge, there have been no reports of the neurometabolic status of the frontoinsular cortex in prematurity survivors.

In our cohort we did not observe any differences in NAA, Cho and mI concentrations in basal ganglia in neither of the hemispheres between the three groups. This could be explained by the low prevalence of cerebral palsy and other major motor dysfunctions in our cohort. We were also unable to find any differences in metabolic spectra of the frontal white matter. We took handedness into consideration–there were only two left-handed children in our cohort.

To assess the neurodevelopmental outcomes in our patients, we utilized two tests that have previously been validated in children at the age of 4. Each test can be used for assessment of different aspects of cognitive function. The Leiter scale is a non-verbal test, that evaluates patient’s IQ. The test samples a wide variety of functions, from memory to nonverbal reasoning and therefore, is considered an adequate tool for assessment of basic aspects of cognitive function. On the contrary, DTVP is used predominantly to evaluate more sophisticated aspects, such as visual perception and visual motor integration.

We found no differences in demographic variables at birth or in the prevalence of prenatal and perinatal complications, including high grade intraventricular haemorrhage (IVH), ROP, BPD and patent ductus arteriosus (PDA) requiring treatment between any of the three groups. In particular, there were no differences between children with mild and children with moderate/severe developmental problems. On the other hand, we found significant disparities in socioeconomic variables between these two groups. More children with mild developmental problems were attending daycare and their fathers were more likely to have obtained higher education compared to children with severe problems. Also, significantly more children with severe problems lived in rural areas. We also found that more children with mild problems had received breastfeeding after birth.

Previous reports from cohort studies suggest that cognitive outcomes of premature children are correlated with environmental factors and parental socio-economic status. Over 25 years ago, Hunt et al.[16] reported that the level of parental education is an important determinant of severity of neurodevelopmental disability. Similarly, Ment et al.[17] found that the improvement of language skills in VLBW children is associated with maternal education and residing in a two-parent household. A study published in 2011 [18] further confirmed these observations by reporting a strong correlation between maternal education, parental marital status and neurodevelopmental outcomes at the age of 24 months. Moreover, Voss et al. suggested that parental education might in fact be the most important predictor of neurodevelopmental outcome in extreme prematurity survivors.[19] Breastfeeding has also been identified as a possible protective factor with regards to neurodevelopment following extreme prematurity in cohort studies, including the EPIPAGE study[20]. Our results seem to partially confirm previous observations, as we found a relationship between paternal education and neurodevelopmental outcome. Parental education, breastfeeding and daycare attendance were the only identifiable factors associated with milder neurodevelopmental impairment in children with abnormal results of DTVP or Leiter test.

NAA has been referred to as a neuronal marker. Studies investigating diseases known to involve neuronal and axonal loss, such as stroke[21], brain tumors[22] of multiple sclerosis (MS) have all shown decrease in NAA concentrations in the affected areas. Moreover, some of them found a correlation between clinical outcomes and NAA concentrations.[23] Even though there have been reports questioning neuronal specificity[24,25] of NAA, it is considered the best surrogate non-invasive marker of neuronal integrity. Choline on the other hand has been reported as a marker of increased cellular membrane turnover. Decreased concentrations of choline were described in hepatic encephalopathy[26] or stroke.[27] Finally, it is unclear whether alterations in myoinositol concentration are significant. It has previously been reported that the concentration of mI is higher in glial cells than by neurons.[28] Therefore it has been proposed as a marker of glial function. We speculate that the lower concentration of all of these metabolites in the left frontoinsular gray matter might reflect a disruption in the integrity of this region, affecting both neuronal and glial function. It might explain a long-term dysfunction of the integrative role of the frontoinsular cortex. Based on our results we propose that neurodevelopmental differences observed in prematurity survivors without macroscopic brain structure abnormalities may be attributed to subtle submicroscopic and metabolic changes. Lower NAA, Cho and mI concentrations in the left frontoinsular gray matter could be related to lower scores in Leiter scale and DTVP and reflect significant neurodevelopmental abnormalities.

To our knowledge, this is the first study investigating cerebral concentrations of several metabolites in the frontoinsular gray matter in prematurity survivors in the long-term perspective. Several previously reported studies investigated metabolic profile of cerebral white matter in adolescent survivors of prematurity, as outlined in the introduction. There is, however, very little long-term data describing metabolism of the gray matter in prematurity survivors.

Our study has several limitations. Firstly, it is based on a cross-sectional design, so the results cannot be used as a tool for predicting neurodevelopmental outcome. It is also very difficult to prove that there is in fact a causal relationship between metabolic differences in left frontoinsular gray matter and neurodevelopmental outcomes. Our group consisted of a relatively small number of individuals, which limits the magnitude of the results that we obtained. Another potential shortcoming is the fact that the study protocol did not include a control group of children born at term. Thus, we were only able to divide our cohort based on the functional results.

In conclusion, our results suggest that long term neurodevelopmental outcomes of prematurely born children without macroscopic brain lesions might be determined by neurometabolic differences in left frontoinsular gray matter. The differences in socioeconomic variables in our cohort, including parental education, demonstrate the potential window for postnatal therapeutic interventions that could improve neurocognitive performance of prematurity survivors.
